# Supplementary material for: Human fingertip regeneration follows clinical phases with distinct proteomic signatures
Source: NPJ Regen Med. 2025 Nov 5;10:51. doi: 10.1038/s41536-025-00441-y (PMC12589496; doi:10.1038/s41536-025-00441-y)
Supplement: Supplementary file 1 — Supplementary Information [file 41536_2025_441_MOESM1_ESM.pdf]

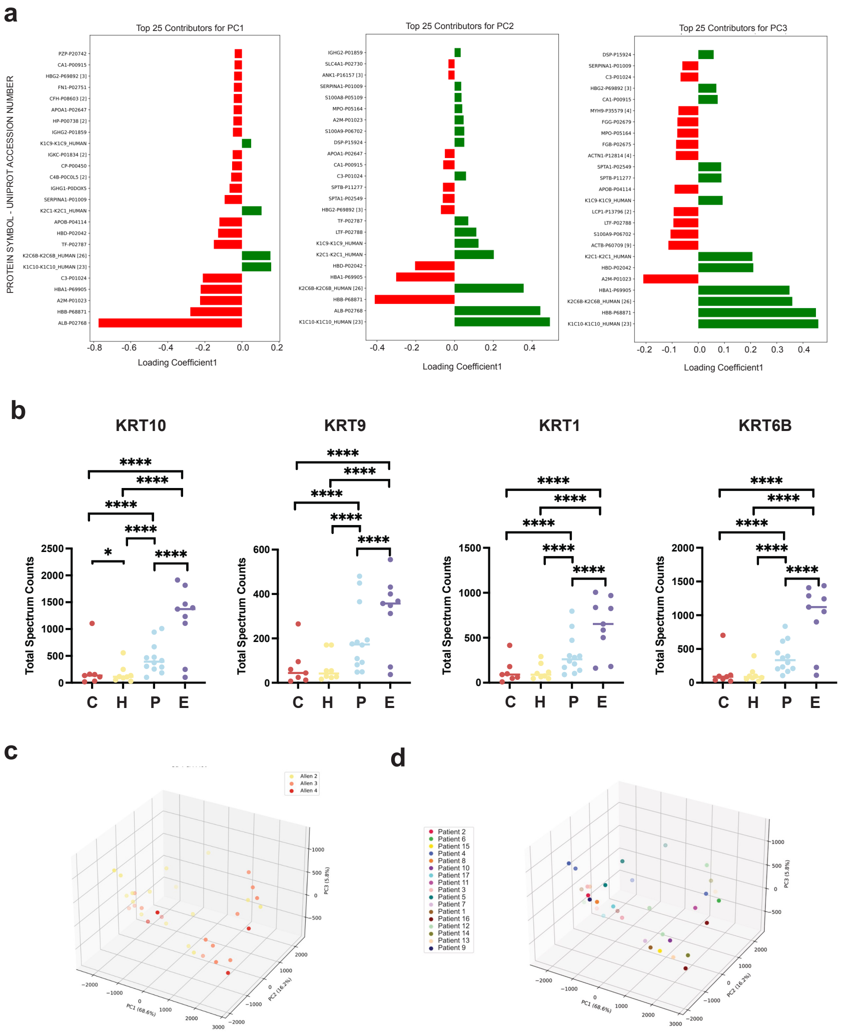
a
Top 25 Contributors for PC1
Top 25 Contributors for PC2
Top 25 Contributors for PC3
PROTEIN SYMBOL - UNIPROT ACCESSION NUMBER
Loading Coefficient1
b
KRT10
KRT9
KRT1
KRT6B
Total Spectrum Counts
C
H
P
E
c
d

Supplementary Fig.1. Protein contribution to PCA analysis.

a. Top 25 protein contributors to the separation of phases in the PCA1, PCA2, and PCA3, plotted by their ID name and number, and their Loading Coefficient. Red indicates a positive coefficient contribution to the principal component, while green indicates a negative coefficient contribution to the principal component.

b. Keratins are a primary contributor to the variation in PC3. KRT10, KRT9, KRT1 and KRT6B were detected by MS/MS analysis. One way ANOVA; significance denoted by ns (p>0.05), *(p≤0.05), **(p≤0.01), ***(p≤0.001), or ****(p≤0.0001).

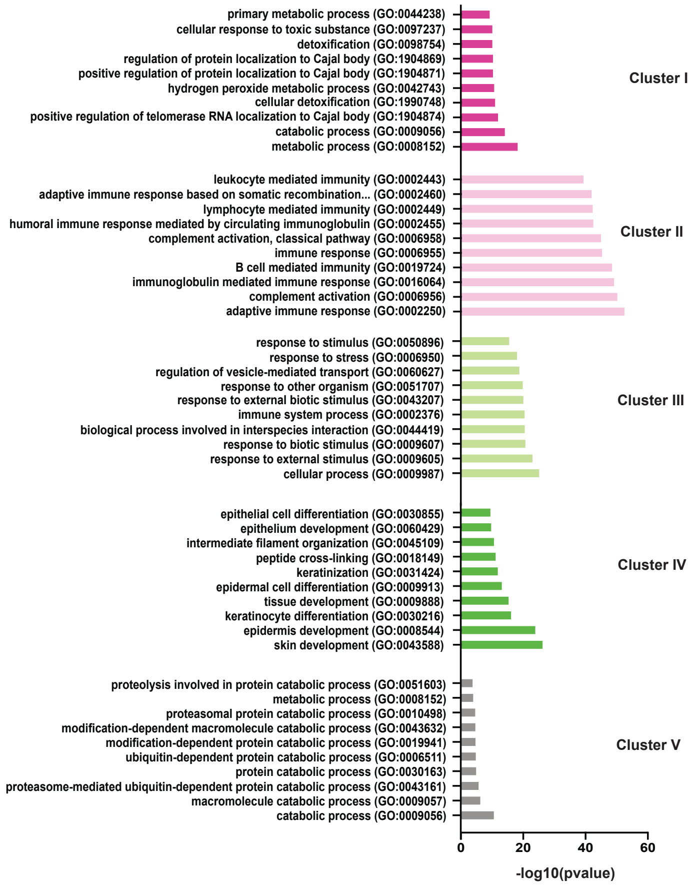

primary metabolic process (GO:0044238)
cellular response to toxic substance (GO:0097237)
detoxification (GO:0098754)
regulation of protein localization to Cajal body (GO:1904869)
positive regulation of protein localization to Cajal body (GO:1904871)
hydrogen peroxide metabolic process (GO:0042743)
cellular detoxification (GO:1990748)
positive regulation of telomerase RNA localization to Cajal body (GO:1904874)
catabolic process (GO:0009056)
metabolic process (GO:0008152)
Cluster I
leukocyte mediated immunity (GO:0002443)
adaptive immune response based on somatic recombination... (GO:0002460)
lymphocyte mediated immunity (GO:0002449)
humoral immune response mediated by circulating immunoglobulin (GO:0002455)
complement activation, classical pathway (GO:0006958)
immune response (GO:0006955)
B cell mediated immunity (GO:0019724)
immunoglobulin mediated immune response (GO:0016064)
complement activation (GO:0006956)
adaptive immune response (GO:0002250)
Cluster II
response to stimulus (GO:0050896)
response to stress (GO:0006950)
regulation of vesicle-mediated transport (GO:0060627)
response to other organism (GO:0051707)
response to external biotic stimulus (GO:0043207)
immune system process (GO:0002376)
biological process involved in interspecies interaction (GO:0044419)
response to biotic stimulus (GO:0009607)
response to external stimulus (GO:0009605)
cellular process (GO:0009987)
Cluster III
epithelial cell differentiation (GO:0030855)
epithelium development (GO:0060429)
intermediate filament organization (GO:0045109)
peptide cross-linking (GO:0018149)
keratinization (GO:0031424)
epidermal cell differentiation (GO:0009913)
tissue development (GO:0009888)
keratinocyte differentiation (GO:0030216)
epidermis development (GO:0008544)
skin development (GO:0043588)
Cluster IV
proteolysis involved in protein catabolic process (GO:0051603)
metabolic process (GO:0008152)
proteasomal protein catabolic process (GO:0010498)
modification-dependent macromolecule catabolic process (GO:0043632)
modification-dependent protein catabolic process (GO:0019941)
ubiquitin-dependent protein catabolic process (GO:0006511)
protein catabolic process (GO:0030163)
proteasome-mediated ubiquitin-dependent protein catabolic process (GO:0043161)
macromolecule catabolic process (GO:0009057)
catabolic process (GO:0009056)
Cluster V
0
20
40
60
-log10(pvalue)

Supplementary Fig. 2. Gene Ontology (GO) enrichment analysis.

Gene ontology (GO) enrichment of Biological Processes was performed using Panther, representing the Top 10 significantly enriched terms for the proteins included in the 5 sub-clusters in Fig. 2f. Terms from the category of Biological Processes were selected using Fisher's Exact test and a Bonferroni correction.

a

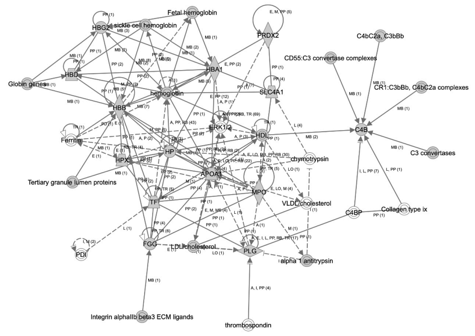
Fetal hemoglobin
sickle cell hemoglobin
PRDX2
HBA1
hemoglobin
SLC4A1
Globin genes
HBB
ERK1/2
HDL
C4B
C4bC2a, C3bBb
CD55:C3 convertase complexes
CR1:C3bBb, C4bC2a complexes
C3 convertases
Ferritin
HPX
HP
APOA1
chymotrypsin
MPO
Tertiary granule lumen proteins
TF
VLDL cholesterol
C4BP
Collagen type ix
FGG
LDL cholesterol
PLG
alpha 1 antitrypsin
Integrin alphaIIb beta3 ECM ligands
thrombospondin

b

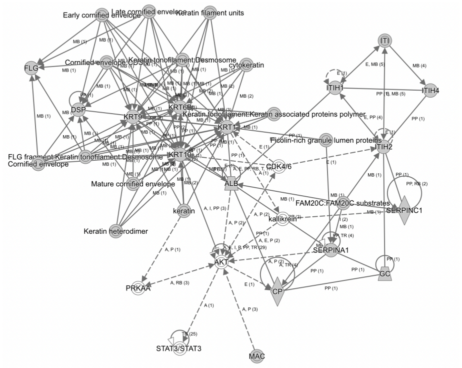
Early cornified envelope
Late cornified envelope
Keratin filament units
FLG
Cornified envelope
Keratin tonofilament:Desmosome
cytokeratin
ITI
ITIH1
ITIH4
DSP
KRT9
Keratin tonofilament:Keratin associated proteins polymer
ITIH2
Ficolin-rich granule lumen proteins
FLG fragment:Keratin tonofilament:Desmosome
Cornified envelope
CDK4/6
ALB
Mature cornified envelope
FAM20C:FAM20C substrates
SERPINC1
keratin
kallikrein
Keratin heterodimer
SERPINA1
AKT
GC
PRKAA
CP
STAT3/STAT3
MAC

Supplementary Fig. 3. Network Diagrams of the DEPs.

IPA Network diagrams showing the highest degree of direct interactions between the 60 DEPs proteins for all phases. **a.** The first network has a prominent presence of blood/immune proteins. **b.** The second network is prominent with components of ECM and its regulators. Direct and indirect interactions are represented as solid and dotted lines. Shapes represent proteins labeled by their name and their interactions supported by at least one reference from the literature (number in parentheses).

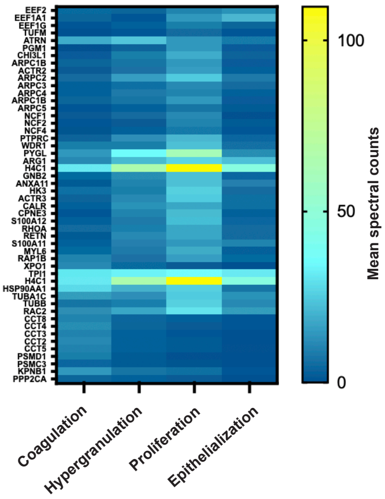

EEF2
EEF1A1
EEF1G
TUFM
ATRN
PGM1
CHI3L1
ARPC1B
ACTR2
ARPC2
ARPC3
ARPC4
ARPC1B
ARPC5
NCF1
NCF2
NCF4
PTPRC
WDR1
PYGL
ARG1
H4C1
GNB2
ANXA11
HK3
ACTR3
CALR
CPNE3
S100A12
RHOA
RETN
S100A11
MYL6
RAP1B
XPO1
TPI1
H4C1
HSP90AA1
TUBA1C
TUBB
RAC2
CCT8
CCT4
CCT3
CCT2
CCT5
PSMD1
PSMC3
KPNB1
PPP2CA
Coagulation
Hypergranulation
Proliferation
Epithelialization
100
50
0
Mean spectral counts

Supplementary Fig. 4. Heatmap of selected cell division and mitosis markers.

Proteins are plotted as average total spectrum counts per phase. Coagulation, Hypergranulation, Proliferation, and Epithelialization.

Coagulation vs Rest

Heme Metabolism

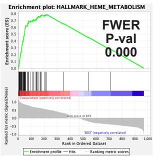

Coagulation

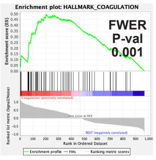

Xenobiotic Metabolism

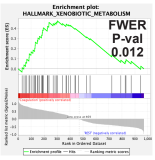

Fatty Acid Metabolism

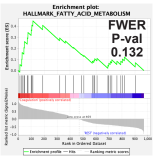

ROS Pathway

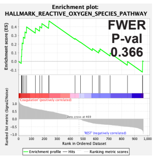

Hypergranulation vs Rest

Coagulation

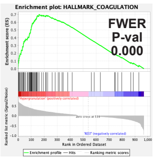

Complement

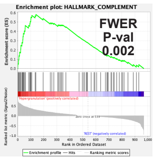

Xenobiotic Metabolism

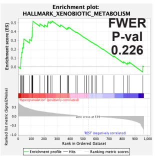

Bile Acid Metabolism

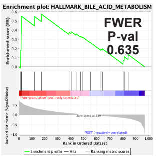

Interferon Gamma Response

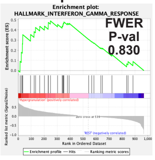

Proliferation vs Rest

Oxidative Phosphorylation

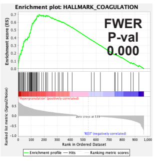

Apical Junction

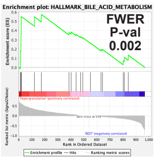

Glycolisis

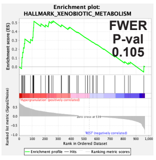

Allograft Rejection

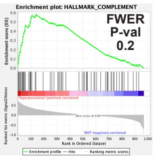

PI3K-AKT-MTOR Signaling

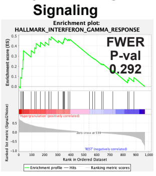

Epithelialization vs Rest

Oxidative Phosphorylation

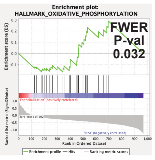

Supplementary Fig. 5. GSEA analysis with top-five hallmark terms for each phase.

Coagulation, Hypergranulation, Proliferation, and Epithelialization in comparison to the other regenerative phases. Graphs depict the positive correlation in red and the negative correlation in blue. FWER *p*-value (P-val) is included for each term graph.

Section/topic	No	CONSORT 2025 checklist item description	Reported on page no.
**Title and abstract**			
Title and structured abstract	1a	Identification as a randomised trial	p 4,14, Ref 30: p. 1, Ref 31, p. 1
	1b	Structured summary of the trial design, methods, results, and conclusions	4, 14, 15 Ref 30: p. 1, Ref 31, p 1
**Open science**			
Trial registration	2	Name of trial registry, identifying number (with URL) and date of registration	4, 15, Ref 30: p. 1
Protocol and statistical analysis plan	3	Where the trial protocol and statistical analysis plan can be accessed	4, 15, Ref 30: p. 1, Ref 31, p 2
Data sharing	4	Where and how the individual de-identified participant data (including data dictionary), statistical code and any other materials can be accessed	4, 15, Ref 30: p. 1
Funding and conflicts of interest	5a	Sources of funding and other support (eg, supply of drugs), and role of funders in the design, conduct, analysis and reporting of the trial	23, Ref 30: p. 1, Ref 31, p 1
	5b	Financial and other conflicts of interest of the manuscript authors	23 Ref 30: p. 1
**Introduction**			
Background and rationale	6	Scientific background and rationale	p. 3-4, Ref 30: p. 2, Ref 31, p. 2
Objectives	7	Specific objectives related to benefits and harms	Ref 30: p. 2,
**Methods**			
Patient and public involvement	8	Details of patient or public involvement in the design, conduct and reporting of the trial	n.a.
Trial design	9	Description of trial design including type of trial (eg, parallel group, crossover), allocation ratio, and framework (eg, superiority, equivalence, non-inferiority, exploratory)	p. 4, Ref 30: p. 2-4, Ref 31, p.2-4
Changes to trial protocol	10	Important changes to the trial after it commenced including any outcomes or analyses that were not prespecified, with reason	Ref. 30, p. 4
Trial setting	11	Settings (eg, community, hospital) and locations (eg, countries, sites) where the trial was conducted	Ref 30: p. 2, Ref 31, p. 3
Eligibility criteria	12a	Eligibility criteria for participants	Ref 30: p. 2, Ref 31, p. 3-4
	12b	If applicable, eligibility criteria for sites and for individuals delivering the interventions (eg, surgeons, physiotherapists)	n.a.
Intervention and comparator	13	Intervention and comparator with sufficient details to allow replication. If relevant, where additional materials describing the intervention and comparator (eg, intervention manual) can be accessed	Ref 30: p. 2-4, Ref 31, p. 2-3

Outcomes	14	Prespecified primary and secondary outcomes, including the specific measurement variable (eg, systolic blood pressure), analysis metric (eg, change from baseline, final value, time to event), method of aggregation (eg, median, proportion), and time point for each outcome	Ref 30: p. 4, Ref 31, p. 4
Harms	15	How harms were defined and assessed (eg, systematically, non-systematically)	Ref 30: p. 2, Ref 31, p. 4-5
Sample size	16a	How sample size was determined, including all assumptions supporting the sample size calculation	Ref 30: p. 4, Ref 31, p. 4
	16b	Explanation of any interim analyses and stopping guidelines	Ref 30: p. 5, Ref 31, p 5
Randomisation:			
Sequence generation	17a	Who generated the random allocation sequence and the method used	Ref 30: p. 5, Ref 31, p. 4
	17b	Type of randomisation and details of any restriction (eg, stratification, blocking and block size)	Ref 30: p. 5, Ref 31, p 4
			**Reported on page no.**
Allocation concealment mechanism	18	Mechanism used to implement the random allocation sequence (eg, central computer/telephone; sequentially numbered, opaque, sealed containers), describing any steps to conceal the sequence until interventions were assigned	Ref 30: p. 5, Ref 31, p 4
Implementation	19	Whether the personnel who enrolled and those who assigned participants to the interventions had access to the random allocation sequence	Ref 30: p. 5, Ref 31, p 4
Blinding	20a	Who was blinded after assignment to interventions (eg, participants, care providers, outcome assessors, data analysts)	n.a.
	20b	If blinded, how blinding was achieved and description of the similarity of interventions	n.a.
Statistical methods	21a	Statistical methods used to compare groups for primary and secondary outcomes, including harms	p. 19-20, Ref 30: p. 5-6, Ref 31, p 5
	21b	Definition of who is included in each analysis (eg, all randomised participants), and in which group	p. 4, Ref 30: p. 5,
	21c	How missing data were handled in the analysis	Ref 30: p. 5, Ref 31, p 5
	21d	Methods for any additional analyses (eg, subgroup and sensitivity analyses), distinguishing prespecified from post hoc	p. 19-20, Ref 30: p. 5, Ref 31, p 5
**Results**			
Participant flow, including flow diagram	22a	For each group, the numbers of participants who were randomly assigned, received intended intervention, and were analysed for the primary outcome	p. 4, Ref 30: p. 5
	22b	For each group, losses and exclusions after randomisation, together with reasons	p. 4, Ref 30: p. 5
Recruitment	23a	Dates defining the periods of recruitment and follow-up for outcomes of benefits and harms	Ref 30: p. 6
	23b	If relevant, why the trial ended or was stopped	Ref 30: p. 6
Intervention and comparator delivery	24a	Intervention and comparator as they were actually administered (eg, where appropriate, who delivered the intervention/comparator, how participants adhered, whether they were delivered as intended (fidelity))	Ref 30: p. 5
	24b	Concomitant care received during the trial for each group	n.a.

Baseline data	25	A table showing baseline demographic and clinical characteristics for each group	Fig.1. 5/6, Ref 30: p. 4
Numbers analysed, outcomes and estimation	26	For each primary and secondary outcome, by group:<br>● the number of participants included in the analysis<br>● the number of participants with available data at the outcome time point<br>● result for each group, and the estimated effect size and its precision (such as 95% confidence interval)<br>● for binary outcomes, presentation of both absolute and relative effect size	p. 4, Ref 30: p. 5ff
Harms	27	All harms or unintended events in each group	Ref 30: p. 6
Ancillary analyses	28	Any other analyses performed, including subgroup and sensitivity analyses, distinguishing pre-specified from post hoc	Ref 30: p. 6
**Discussion**			
Interpretation	29	Interpretation consistent with results, balancing benefits and harms, and considering other relevant evidence	p. 8-13, Ref 30: p. 11
Limitations	30	Trial limitations, addressing sources of potential bias, imprecision, generalisability, and, if relevant, multiplicity of analyses	p.12, 13, Ref 30: p. 11

Citation: Hopewell S, Chan AW, Collins GS, Hróbjartsson A, Moher D, Schulz KF, et al. CONSORT 2025 Statement: updated guideline for reporting randomised trials. BMJ. 2025; 388:e081123. https://dx.doi.org/10.1136/bmj-2024-081123
© 2025 Hopewell et al. This is an Open Access article distributed under the terms of the Creative Commons Attribution License (https://creativecommons.org/licenses/by/4.0/), which permits unrestricted use, distribution, and reproduction in any medium, provided the original work is properly cited.

*We strongly recommend reading this statement in conjunction with the CONSORT 2025 Explanation and Elaboration and/or the CONSORT 2025 Expanded Checklist for important clarifications on all the items. We also recommend reading relevant CONSORT extensions. See www.consort-spirit.org.

Supplementary Data 1_Total Spectral Count

Supplementary Data 2_ Statistics on TSC

Supplementary Data 3_ 5 clusters in Fig 2

Supplementary Data 4_ Statistics 60 Proteins

Supplementary Data 5__60DEPs_coreA_networks

Supplementary Data 6_ IPA canonical Pathways

Supplementary Data 7_ IPA Diseases and Functions

Supplementary Data 8_ IPA Canonical Pathways Statistics

Supplementary Data 9_ Upstream regulators and targets

Supplementary Data 10_ Gprofiler

Supplementary Data 11_GSEA_ranked_gene_list
